# Association between vegetarian diets and cardiovascular risk factors in non-Hispanic white participants of the Adventist Health Study-2

**DOI:** 10.1017/jns.2019.1

**Published:** 2019-02-21

**Authors:** Seiji Matsumoto, W. Lawrence Beeson, David J. Shavlik, Gina Siapco, Karen Jaceldo-Siegl, Gary Fraser, Synnove F. Knutsen

**Affiliations:** 1 Center for Nutrition, Healthy Lifestyle, and Disease Prevention, School of Public Health, Loma Linda University, Loma Linda, CA 92354, USA; 2 Adventist Health Study-2, School of Public Health, Loma Linda University, Loma Linda, CA 92354, USA

**Keywords:** Cardiovascular risk factors, Diets, Adventist Health Study-2, Vegetarian dietary patterns, Lipids, Disease prevalence, AHS-2, Adventist Health Study-2, BP, blood pressure, Bio-MRS, Biologic Manifestations of Religion Study, DBP, diastolic blood pressure, DM, diabetes mellitus, EPIC, European Prospective Investigation into Cancer and Nutrition, FBG, fasting blood glucose, HDL-C, HDL-cholesterol, HR, hazard ratio, LDL-C, LDL-cholesterol, LOV, lacto-ovo-vegetarian, MDS, Mediterranean Diet Score, NV, non-vegetarian, PR, prevalence ratio, PV, pesco-vegetarian, SBP, systolic blood pressure, TC, total cholesterol, VG, vegan, WC, waist circumference

## Abstract

The association between dietary patterns and CVD risk factors among non-Hispanic whites has not been fully studied. Data from 650 non-Hispanic white adults who participated in one of two clinical sub-studies (about 2 years after the baseline) of the Adventist Health Study-2 (AHS-2) were analysed. Four dietary patters were identified using a validated 204-item semi-quantitative FFQ completed at enrolment into AHS-2: vegans (8·3 %), lacto-ovo-vegetarians (44·3 %), pesco-vegetarians (10·6 %) and non-vegetarians (NV) (37·3 %). Dietary pattern-specific prevalence ratios (PR) of CVD risk factors were assessed adjusting for confounders with or without BMI as an additional covariable. The adjusted PR for hypertension, high total cholesterol and high LDL-cholesterol were lower in all three vegetarian groups. Among the lacto-ovo-vegetarians the PR were 0·57 (95 % CI 0·45, 0·73), 0·72 (95 % CI 0·59, 0·88) and 0·72 (95 % CI 0·58, 0·89), respectively, which remained significant after additionally adjusting for BMI. The vegans and the pesco-vegetarians had similar PR for hypertension at 0·46 (95 % CI 0·25, 0·83) and 0·62 (95 % CI 0·42, 0·91), respectively, but estimates were attenuated and marginally significant after adjustment for BMI. Compared with NV, the PR of obesity and abdominal adiposity, as well as other CVD risk factors, were significantly lower among the vegetarian groups. Similar results were found when limiting analyses to participants not being treated for CVD risk factors, with the vegans having the lowest mean BMI and waist circumference. Thus, compared with the diet of NV, vegetarian diets were associated with significantly lower levels of CVD risk factors among the non-Hispanic whites.

The leading cause of death among non-Hispanic whites in the USA is heart disease, comprising close to 23·7 % of total deaths in 2015^(^^1^^)^. For the same racial group, 20 years and older, the 2011–2014 prevalence of the broader CVD category is 37·7 and 35·1 % in men and women, respectively^(^^2^^)^. Apart from our study among non-Hispanic blacks in the Adventist Health Study-2 (AHS-2) study^(^^3^^)^ and three sub-studies among AHS-2 participants^(^^4^^–^^6^^)^, very few other papers are available on the association between plant-based diets and outcomes^(^^7^^–^^12^^)^. These studies have reported lower levels of both CVD risk factors (hypertension, obesity, abdominal adiposity, fasting blood glucose (FBG)) as well as prevalence of dyslipidaemia, CVD and type 2 diabetes mellitus (DM) among vegetarians compared with non-vegetarians (NV)^(^^3^^–^^8^^)^. The European Prospective Investigation into Cancer and Nutrition study (EPIC-Oxford) in England and Scotland (*n*   44 561) found, among a subgroup of 1546 participants, a 32 % lower risk of IHD among vegetarians than among NV^(^^9^^)^. The difference was attributed to a possible protective effect of the vegetarian diet on non-HDL-cholesterol (HDL-C) levels and systolic blood pressure (SBP) when compared with the NV diet, with non-HDL-C being 3·97 (95 % CI 3·84, 4·10) *v.* 4·42 (95 % CI 4·36, 4·47) mmol/l and SBP being 130·7 (95 % CI 128·4, 133·1) *v.* 134·0 (95 % CI 133·0, 134·9) mmHg, respectively^(^^9^^)^. There were similar findings in the Indian Migration Study^(^^10^^)^ where dietary patterns were chosen based on religious faith. Compared with the NV, the vegetarians had lower levels of FBG, total cholesterol (TC), LDL-cholesterol (LDL-C), TAG, and lower diastolic blood pressure (DBP)^(^^10^^)^. Findings from randomised controlled studies in various settings are in line with these findings^(^^11^^,^^12^^)^. In a spectrum of dietary patterns from an omnivore to a more plant-based diet up to the one devoid of any animal products, it appears that the more plant-based the diet is, the lower the all-cause mortality^(^^13^^)^, CVD risk factors^(^^14^^)^ and CVD mortality^(^^15^^)^. The differing dietary patterns were associated with fatty acid intake^(^^16^^)^, of which plant-based and seafood long-chain *n*-3 PUFA have been linked with lower CVD risk factors such as lower TAG, blood pressure (BP) and resting heart rate^(^^17^^)^ and reduced risk of fatal CHD^(^^18^^)^.

While Fraser *et al*.^(^^3^^)^ reported on the advantages of a vegetarian diet on CVD risk factors among the black AHS-2 adults, the association between various dietary patterns and CVD risk factors among the white members of the AHS-2 has not been reported. Thus, the aim of the present cross-sectional study was to describe the association between self-reported dietary patterns at AHS-2 enrolment and clinical CVD risk factor data collected 1–3 years later among non-Hispanic white participants. Similar to a few studies of this association among vegans (VG) as a distinct group^(^^3^^,^^5^^)^ we included three separate vegetarian dietary patterns, namely VG, lacto-ovo-vegetarian (LOV) and pesco-vegetarian (PV), to compare with NV.

## Methods

### Study population

The recruitment of the AHS-2 as well as its sub-studies are described in detail elsewhere^(^^19^^–^^21^^)^. Briefly, non-Hispanic white and black Adventists, aged 30 years and older, were invited to participate and enrolment took place between 2002 and 2007. A total of about 96 000 Adventists from all US states as well as five provinces in Canada were included and of these only 7·8 % were of other race/ethnicities. Non-Hispanic white Adventists who had participated in one of two clinical sub-studies of the AHS-2 were selected for this study. Participants for the calibration study were randomly selected from the present AHS-2 baseline cohort and, for the second clinic, older AHS-2 participants living in southern California were invited to the clinics of the Biologic Manifestations of Religion Study (Bio-MRS), a sub-study of Biopsychosocial Religion and Health Study^(^^19^^)^. A total of 730 white non-Hispanic adults participated in one of these two clinics and biological measurements from these two clinics as well as dietary, demographic and medical history information from the AHS-2 baseline questionnaire (http://www.llu.edu/pages/health/documents/ahs-2.pdf) were used for this study. Fasting blood samples were collected at each of the two clinics and these are used for this analysis. Biometrics and percentage body fat, measured by bioelectrical impedance, were also measured at the clinics.

Exclusion criteria were applied to enrolees’ data with missing information on essential variables such as dietary pattern, BMI, education, BP, waist circumference (WC), exercise, smoking and alcohol use history, FBG and lipid profile. Those data which contained missing information of any of the above variables were excluded from the analytic sample.

### Data collection/outcomes

#### Dietary data

At enrolment, participants in the AHS-2 completed a mailed, self-administered 204-item semi-quantitative FFQ which was validated within 1–2 years by the calibration study^(^^21^^)^. Based on this FFQ, participants were classified into five dietary pattern groups^(^^20^^–^^22^^)^. For the present study, we excluded a small group who ate meat more than once per month, but less than once a week. Thus we are presenting results for four dietary patterns, including VG who ate animal products never or rarely (<1/month), LOV who ate dairy products and eggs, but meat/fish was consumed <1/month, PV who ate fish >1/month, but other meats <1/month, and NV who ate meat products more than once/week^(^^23^^)^.

#### Clinical data

Biological measurements were collected in the clinic sub-studies which were conducted 1–3 years after enrolment into the AHS-2. The purpose of the calibration study was to validate dietary and other information from the baseline AHS-2 questionnaire^(^^24^^)^ and that of the Bio-MRS was to examine biological indicators of allostatic load^(^^19^^,^^24^^)^. The same clinic protocol was used for these two clinic studies and included a fasting blood sample, BP, and anthropometric measurements (weight, height and WC), and assessment of FBG and lipid panel (TC, HDL-C and TAG). FBG, TC, HDL-C and TAG were measured using a Cholestech LDX analyser (Cholestech Corp.). LDL-C was computed using the Friedewald formula^(^^25^^)^. Non-HDL-C was computed subtracting HDL-C from TC. After a 10-min rest, SBP and DBP were measured three times consecutively with 1-min intervals using an automatic device (Omron HEM-747IC; Omron Healthcare, Inc.). For analyses, the 2nd and the 3rd readings were averaged. Weight was measured using a digital scale (Tanita BF-350 Body Composition Analyser; Tanita UK Ltd) without shoes or heavy outer garments. Height was measured without shoes to the nearest millimetre using a portable stadiometer (INVICTA Height Measure, reference no. 0955; Invicta Plastics Ltd). BMI was computed as weight (kg)/height^2^ (m^2^). Waist circumference was measured in millimetres at the mid-point between the lower rib and the upper margin of the iliac crest using a plastic tape. Exercise was computed as time (min/week) spent in walking, running, or jogging, or in any vigorous aerobic activity reported in the baseline AHS-2 questionnaire. The baseline questionnaire also provided demographic, medical history and other lifestyle information such as smoking and alcohol use. The detailed data collection method, validation of the AHS-2 FFQ and description of the clinical data (the calibration study and the Bio-MRS) are reported elsewhere^(^^19^^,^^21^^,^^22^^,^^26^^)^.

### Ethical standards

The study was approved by the Loma Linda University Institutional Review Board.

### Statistical analysis

Descriptive analyses were performed on selected demographic and anthropometric factors, smoking (ever/never) and alcohol history (ever/never), according to the four dietary patterns: VG, LOV, PV and NV using *χ*^2^ analysis (categorical variables), one-way ANOVA for mean age across the dietary patterns, and the Kruskal–Wallis test for medians of time spent on exercises, BMI and WC.

Because of the high occurrence of our outcomes and the known OR inflation by logistic regression when estimating RR of common disease when confounders are present, it is becoming common to choose alternative estimation methods such as log-binomial or Poisson regression, and the latter is preferred^(^^27^^–^^29^^)^. Therefore, to estimate the prevalence ratios (PR) and CI of the CVD risk factors (hypertension, DM, hyperlipidaemias and low HDL-C, high abdominal adiposity and obesity) by dietary patterns, using NV as the reference, we used modified Poisson regression analyses (Poisson regression with robust error variance)^(^^30^^,^^31^^)^ with an *a priori* model.

*A priori* covariables included age, sex (as appropriate), education (as this is a documented marker of socio-economic status in the Adventist population^(^^32^^)^ and in others^(^^33^^,^^34^^)^), marital status, exercise amount (min/week), history of smoking (ever/never), alcohol drinking history (ever/never), a study indicator (calibration or Bio-MRS), and BMI as appropriate for the initial model. Risk factor levels were defined the same as in our previous publication among the black AHS-2 members^(^^3^^)^: hypertension (SBP ≥ 140 mmHg and/or DBP ≥ 90 mmHg or taking antihypertensive medications); DM (fasting plasma glucose ≥126 mg/dl (or 7 mmol/l) or taking antiglycaemic agents); high TC (taking cholesterol-lowering medication or having levels >200 mg/dl (5·17 mmol/l)); and high LDL-C (taking cholesterol-lowering medication or having levels ≥130 mg/dl (3·36 mmol/l)); low HDL-C (<40 mg/dl (1·03 mmol/l) (males) or <50 mg/dl (1·29 mmol/l) (females)); and high TAG (taking lipid-lowering medication or having levels >150 mg/dl (1·69 mmol/l))^(^^35^^)^; obesity (BMI ≥ 30·0 kg/m^2^); abdominal adiposity (WC >88 cm in females and >102 cm in males).

The potential interaction between age and the main explanatory variable (i.e. dietary pattern) was tested in all models used to estimate adjusted PR.

Self-reported medications for treatment of hypertension, DM, hypercholesterolaemia or dyslipidaemia were ascertained by a cardiologist/internist (G. F.). The adjusted means of each risk factor as well as lipid ratios including TC:HDL-C, TAG:HDL-C and apoB:apoA-I by dietary pattern were computed among participants who did not take any medications for each particular risk factor. A generalised linear model was used for these computations adjusting for age, sex, education, marital status, exercise, history of ever smoking and/or ever drinking, BMI wherever appropriate, and an indicator variable for sub-study. The sex-specific apoB:apoA-I ratios were estimated according to the regression formulas used by Walldius & Jungner^(^^36^^)^ and then combined for both sexes. The adjusted means were computed both with and without BMI in the model. Comparisons were made between each vegetarian group *v.* NV by pairwise contrast. Sub-group analyses by sex to compute adjusted PR and least square means were also performed as necessary.

A small number of missing dietary variable data (3–7 %) which were used for determination of the dietary patterns were imputed by a guided multiple imputation method^(^^4^^,^^37^^)^. Statistical analyses were carried out using the SAS 9.4 statistical software package (SAS Institute Inc.).

## Results

A total of 730 non-Hispanic white Adventists participated and their blood was drawn in one of the two study clinics: 407 from the calibration study, and 243 from the Bio-MRS. After applying the exclusion criteria, which eliminated eighty, a total of 650 adults, 397 women and 253 men, were included in the present study. Of these, 37·1 % were NV and 62·9 % were vegetarians (8·3 % VG, 44·3 % LOV and 10·3 % PV) (Table 1). The VG, LOV and the PV were older than the NV (64·8, 62·6, 64·7, and 59·7 years, respectively; *P* = 0·004; Table 1). Except for sex, marital status, DM, low HDL-C and high TAG, the demographic characteristics and other risk factor variables varied significantly across the dietary patterns. A higher proportion of LOV and PV were college graduates compared with the VG or NV (61·1 and 56·7 % *v.* 44·4 and 46·9 %, respectively).

**Table 1.** Characteristics of selected covariables, cardiovascular risk factors and prevalent cardiovascular events by dietary patterns among non-Hispanic white participants in the Adventist Health Study-2 (Numbers of participants and percentages; mean values and standard deviations; medians and interquartile ranges (IQR) indicated with 1st quartile and 3rd quartile values)

	Vegan (*n* 54; 8·3 %)		Lacto-ovo-vegetarian (*n* 288; 44·3 %)		Pesco-vegetarian (*n* 67; 10·3 %)		Non-vegetarian (*n* 241; 37·1 %)		Total (*n* 650)
	*n*	%	*n*	%	*n*	%	*n*	%	P*
Age (years)									
<50	4	7·4	41	14·2	10	14·9	38	15·8	0·003
50–59	10	18·5	68	23·6	5	7·5	62	25·7	
60–69	17	31·5	56	19·4	20	29·9	70	29·1	
≥70	23	42·6	123	42·7	32	47·8	71	29·5	
Mean	64·8		62·6		64·7		59·7		0·004
sd	10·8		14·0		13·7		12·3	
Sex									
Women	37	68·5	176	61·1	39	58·2	145	60·2	0·67
Men	17	31·5	112	38·9	28	41·8	96	39·8	
Marital status†									
Married/married under common law	45	83·3	238	83·2	49	74·2	191	80·3	0·37
Single/divorced/separated/widowed	9	16·7	48	16·8	17	25·8	47	19·7	
Education									
High school or below	15	27·8	36	12·5	15	22·4	48	19·9	0·005
Some college	15	27·8	76	26·4	14	20·9	80	33·2	
Bachelors and above	24	44·4	176	61·1	38	56·7	113	46·9	
Physical activity (min/week)									
Median	140·0		75·0		90·0		75·0		0·004
IQR	70·0, 225·0		32·5, 140·0		5·0, 150·0		15·0, 150·0	
BMI (kg/m^2^)									
<25	38	70·4	167	58·0	30	44·8	64	26·6	<0·0001
25–29·9	11	20·4	89	30·9	30	44·8	98	40·7	
≥30	5	9·3	32	11·1	7	10·5	79	32·8	
Median	22·9		24·3		25·7		27·0		<0·0001
IQR	20·8, 26·4		22·1, 27·0		22·9, 27·3		24·9, 31·1	
Waist circumference (cm)									
Median	81·0		88·5		90·6		96·5		<0·0001
IQR	77·5, 91·9		78·7, 97·4		82·6, 99·7		86·4, 106·3		
Smoking (ever smoked)									
No	46	85·2	272	94·4	60	89·6	199	82·6	0·0002
Yes	8	14·8	16	5·6	7	10·4	42	17·4	
Alcohol (ever drink)									
No	40	74·1	236	81·9	47	70·1	141	58·5	<0·0001
Yes	14	25·9	52	18·1	20	29·9	100	41·5	
Hypertension									
Yes	17	31·5	99	34·8	24	35·8	110	45·6	0·035
No	37	68·5	189	65·6	43	64·2	131	54·4	
Diabetes†									
Yes	0	0·0	11	4·2	2	3·2	15	7·0	0·148‡
No	53	100·0	250	95·8	60	96·8	198	93·0	
High total cholesterol†									
Yes	23	43·4	109	40·1	31	49·2	127	54·0	0·016
No	30	56·6	163	59·9	32	50·8	108	46·0	
High LDL-cholesterol†									
Yes	21	40·4	94	35·0	24	38·1	116	50·0	0·007
No	31	59·6	175	65·1	39	61·9	116	50·0	
Low HDL-cholesterol									
Yes	28	51·9	160	55·6	33	49·3	113	46·9	0·253
No	26	48·2	128	44·4	34	50·8	128	53·1	
High TAG†									
Yes	15	28·9	95	35·5	20	32·3	93	40·4	0·326
No	37	71·2	173	64·6	42	67·7	137	59·6	
Obesity									
Yes	5	9·3	32	11·1	7	10·5	79	32·8	<0·001
No	49	90·7	256	88·9	60	89·6	162	67·2	
Abdominal adiposity									
Yes	11	20·4	81	28·1	25	37·3	129	53·5	<0·001
No	43	79·6	207	71·9	42	62·7	112	46·5	
Overall CVD									
Yes	14	25·9	88	30·6	19	28·4	72	30·0	0·912
No	40	74·1	200	69·4	48	71·6	169	70·1	
Myocardial infarction									
Yes	1	1·9	12	4·2	0	0·0	16	6·6	0·078‡
No	53	98·2	276	95·8	67	100·0	225	93·4	
Stroke									
Yes	1	1·9	3	1·0	0	0·0	5	2·1	0·529‡
No	53	98·2	285	99·0	67	100·0	236	97·9	
Small stroke									
Yes	1	1·9	11	3·8	2	3·0	6	2·5	0·822‡
No	53	98·2	277	96·2	65	97·0	235	97·5	
Angina pectoris									
Yes	2	3·7	9	3·1	0	0·0	12	5·0	0·227‡
No	52	96·3	279	96·9	67	100·0	229	95·0	
Congestive heart failure									
Yes	2	3·7	7	2·4	1	1·5	5	2·1	0·879‡
No	52	96·3	281	97·6	66	98·5	236	97·9	
Stent/bypass									
Yes	4	7·4	12	4·2	2	3·0	10	4·2	0·662‡
No	50	92·6	276	95·8	65	97·0	231	95·9	
Carotid surgery									
Yes	2	3·7	0	0·0	0	0·0	1	0·4	0·014‡
No	52	96·3	288	100·0	67	100·0	240	99·6	
Sub-study									
Calibration	44	81·5	157	54·5	38	56·7	168	69·7	<0·0001
Bio-MRS	10	18·5	131	45·5	29	43·3	73	30·3	

Bio-MRS, Biological Manifestations of Religion Study.

* *P* values test differences between means of dietary patterns or the null hypotheses of no association between the named variable and dietary pattern.

† Does not add up to *n* 650 due to missing values.

‡ Fisher's exact test was used.

The prevalence of CVD or related surgeries was relatively low across all dietary patterns and only the proportion of participants with previous carotid surgery was significantly different (*P* = 0·014) across the dietary patterns (Table 1). However, there were only three cases of carotid surgery in the study population, two among the VG and one among the NV.

Compared with the NV, the estimated PR of major prevalent CVD risk factors in the three vegetarian dietary patterns are summarised in Table 2. Regardless of whether we adjusted for BMI or not, the PR for prevalent hypertension, high TC, high LDL-C, obesity and abdominal adiposity were lower in the LOV compared with the NV after adjusting for a number of covariables. This was the case for both sexes though not all were statistically significant (not shown except for obesity and abdominal adiposity). The VG and PV had similar or lower PR as those observed for the LOV compared with the NV. However, not all values reached statistical significance. This was also true when additionally adjusting for BMI.

**Table 2.** Poisson regression* analyses to compare prevalence ratios (PR) of major cardiovascular risk factors between dietary patterns among non-Hispanic white participants in the Adventist Health Study-2 at baseline (Prevalence ratios and 95 % confidence intervals)

	Vegan (reference non-vegetarian)			Lacto-ovo-vegetarian (reference non-vegetarian)			Pesco-vegetarian (reference non-vegetarian)		
	*n*	PR	95 % CI	*n*	PR	95 % CI	*n*	PR	95 % CI
Not adjusted for BMI									
Hypertension†‡	54	0·46	0·25, 0·83	288	0·57	0·45, 0·73	67	0·62	0·42, 0·91
Systolic hypertension†‡	54	0·48	0·26, 0·87	288	0·58	0·45, 0·75	67	0·62	0·41, 0·93
Diastolic hypertension§	54	0·47	0·27, 0·83	288	0·60	0·46, 0·78	67	0·43	0·26, 0·72
Diabetes‖	53	–	–	261	0·62	0·29, 1·34	62	0·48	0·10, 2·27
High total cholesterol¶	53	0·77	0·55, 1·07	272	0·72	0·59, 0·88	63	0·87	0·66, 1·16
High LDL-cholesterol¶	52	0·77	0·54, 1·12	269	0·72	0·58, 0·89	63	0·74	0·53, 1·05
Low HDL-cholesterol¶	54	1·14	0·85, 1·54	288	1·22	1·03, 1·46	67	1·06	0·80, 1·41
High TAG¶	52	0·66	0·42, 1·05	268	0·89	0·71, 1·13	62	0·80	0·54, 1·19
Obesity**	54	0·31	0·13, 0·74	288	0·37	0·26, 0·55	67	0·34	0·17, 0·70
Obesity, females**	37	0·39	0·15, 1·04	176	0·48	0·32, 0·74	39	0·33	0·13, 0·83
Obesity, males**	17	0·18	0·03, 1·31	112	0·22	0·09, 0·50	28	0·36	0·12, 1·06
Abdominal adiposity§**	54	0·39	0·23, 0·67	288	0·55	0·44, 0·69	67	0·71	0·51, 0·99
Abdominal adiposity, females§**	37	0·42	0·23, 0·75	176	0·57	0·45, 0·74	39	0·67	0·45, 0·99
Abdominal adiposity, males**	17	0·32	0·09, 1·17	112	0·53	0·33, 0·85	28	0·83	0·45, 1·51
Adjusted for BMI									
Hypertension†	54	0·58	0·31, 1·06	288	0·66	0·52, 0·85	67	0·69	0·47, 1·02
Systolic hypertension†‡	54	0·59	0·32, 1·09	288	0·67	0·52, 0·87	67	0·68	0·45, 1·03
Diastolic hypertension§	54	0·62	0·35, 1·10	288	0·72	0·55, 0·95	67	0·49	0·30, 0·79
Diabetes‖	53	–	–	261	0·92	0·42, 2·00	62	0·61	0·13, 2·91
High total cholesterol¶	53	0·81	0·58, 1·14	272	0·75	0·62, 0·92	63	0·90	0·68, 1·20
High LDL-cholesterol¶	52	0·85	0·58, 1·24	269	0·76	0·61, 0·95	63	0·78	0·55, 1·10
Low HDL-cholesterol¶	54	1·30	0·96, 1·75	288	1·33	1·12, 1·59	67	1·13	0·85, 1·49
High TAG¶	52	0·81	0·51, 1·29	268	1·02	0·81, 1·29	62	0·88	0·59, 1·33

* A separate Poisson regression model was run for each risk factor, in each case adjusted for age, sex (as appropriate), education, marital status, physical activity (min/week), history of ever smoking, history of ever drinking alcohol, sub-study indicator and BMI as indicated.

† Hypertension is systolic blood pressure ≥ 140 mmHg and/or diastolic blood pressure ≥ 90 mmHg or taking medications for high blood pressure.

‡ PR computed at the mean age of 61·9 years.

§ PR for vegetarian *v.* non-vegetarian computed at the mean age of 61·9 years.

‖ Diabetes is fasting blood sugar ≥7 mmol/l (or 126 mg/dl) or taking glucose-lowering medications.

¶ High total cholesterol is ≥5·17 mmol/l (or 200 mg/dl) and high LDL-cholesterol is ≥3·36 mmol/l (or 130 mg/dl) or taking cholesterol-lowering medications in either case; low HDL-cholesterol is <1·03 mmol/l (or 40 mg/dl) (males) or <1·29 mmol/l (or 50 mg/dl) (females); high TAG is >1·69 mmol/l (or 150 mg/dl) or taking statin-like medications.

** Obesity is BMI ≥30·0 kg/m^2^; abdominal adiposity is waist circumference >88 cm in females and >102 cm in males.

### Hypertension and dietary patterns

A total of 250 participants were classified as hypertensive and 140 of these were using anti-hypertensive medications. In the multivariable Poisson regression, the PR for having prevalent hypertension were significantly lower among the three vegetarian groups (PR = 0·46 (95 % CI 0·25, 0·83), 0·57 (95 % CI 0·45, 0·73) and 0·62 (95 % CI 0·42, 0·91) for the VG, LOV and PV, respectively, compared with the NV; Table 2). When also adjusting for BMI, the estimates were slightly weakened, but remained significant for the LOV. Compared with the NV, the adjusted mean SBP and DBP values were lower among the VG, LOV and PV, with 7·1, 6·1 and 4·2 mmHg lower values, respectively, for SBP, and with 5·9, 4·0 and 3·1 mmHg, respectively, for DBP (Table 3). The values for the PV were the least different and only DBP reached statistical significance (Table 3). When also adjusting for BMI, the lower values of DBP among VG and LOV persisted, but were attenuated and non-significant for the PV.

**Table 3.** Adjusted mean levels of risk factors* by dietary patterns among non-Hispanic white participants in the Adventist Health Study-2 at baseline

	Vegan	Lacto-ovo- vegetarian	Pesco- vegetarian	Non- vegetarian	*P*†: vegan *v*. non-vegetarian	*P*†: lacto-ovo-vegetarian *v*. non-vegetarian	*P*†: pesco-vegetarian *v*. non-vegetarian
Not adjusted for BMI							
** **Systolic BP (mmHg)	118·0	119·0	120·9	125·1	0·02	0·0009	0·12
** **Diastolic BP (mmHg)	69·3	71·2	72·1	75·2	0·0004	0·0001	0·04
** **FBG (mmol/l)	4·77	5·00	4·91	5·24	0·0007	0·004	0·01
** **TC (mmol/l)	4·80	4·75	4·91	5·10	0·05	0·0002	0·18
** **LDL-C (mmol/l)	3·00	2·91	3·04	3·11	0·44	0·01	0·56
** **HDL-C (mmol/l)	1·20	1·22	1·25	1·26	0·32	0·20	0·79
** **Non-HDL-C (mmol/l)	3·61	3·53	3·66	3·83	0·19	0·002	0·26
** **TC:HDL-C ratio	4·29	4·36	4·43	4·41	0·65	0·73	0·95
** **TAG (mmol/l)	1·33	1·40	1·37	1·59	0·06	0·03	0·08
** **TAG:HDL-C ratio	2·90	3·28	3·00	3·46	0·25	0·54	0·30
** **apoB:apoA-I ratio‡	0·73	0·71	0·73	0·75	0·40	0·02	0·56
** **BMI (kg/m^2^), both	24·1	25·3	26·0	28·2	<0·0001	<0·0001	0·002
** **BMI (kg/m^2^), females	24·7	25·4	25·7	28·2	0·0008	<0·0001	0·01
** **BMI (kg/m^2^), males	22·9	25·2	26·5	28·1	<0·0001	<0·0001	0·08
** **WC (cm), both	87·4	90·5	91·8	97·6	0·0002	<0·0001	0·02
** **WC (cm), females	85·8	86·4	86·7	94·7	0·03	0·0006	0·04
** **WC (cm), males	88·9	96·1	100·5	102·5	<0·0001	0·0003	0·46
Adjusted for BMI							
** **Systolic BP (mmHg)	119·8	120·0	120·8	123·2	0·24	0·08	0·37
** **Diastolic BP (mmHg)	70·5	71·9	72·0	73·9	0·03	0·04	0·20
** **FBG (mmol/l)	4·85	5·04	4·92	5·17	0·02	0·14	0·05
** **TC (mmol/l)	4·80	4·75	4·91	5·10	0·07	0·0004	0·20
** **LDL-C (mmol/l)	3·01	2·91	3·04	3·11	0·47	0·02	0·58
** **HDL-C (mmol/l)	1·16	1·20	1·24	1·30	0·03	0·008	0·29
** **Non-HDL-C (mmol/l)	3·66	3·55	3·67	3·79	0·44	0·02	0·43
** **TC:HDL-C ratio	4·42	4·42	4·46	4·30	0·68	0·50	0·54
** **TAG (mmol/l)	1·42	1·45	1·39	1·51	0·53	0·44	0·32
** **TAG:HDL-C ratio	3·19	3·42	3·06	3·20	0·98	0·44	0·73
** **apoB:apoA-I ratio‡	0·73	0·71	0·73	0·75	0·43	0·03	0·58

BP, blood pressure; FBG, fasting blood glucose; TC, total cholesterol; LDL-C, LDL-cholesterol; HDL-C, HDL-cholesterol; WC, waist circumference.

* Among participants not on medication for that particular risk factor (applies to hypertension, diabetes and lipids), adjusted for age, sex (as appropriate), marital status, education, history of ever smoking, history of ever drinking, physical activity, sub-study indicator and BMI as indicated.

† *P* values test the null hypotheses of no difference between the pairs of dietary groups indicated.

‡ The apoB:apoA-I ratio was estimated by regression formula for each sex which was proposed by Walldius & Jungner^(^^36^^)^ and combined for both sexes.

### Diabetes mellitus and dietary patterns

A total of twenty-eight participants were self-reported physician diagnosed as having DM and, of these, twenty-two were taking diabetic medications. The unadjusted prevalence of DM was higher among NV, with 7·0 %, compared with 0·0, 4·2 and 3·2 % among the VG, LOV and PV, respectively, but the difference was not significant (*P* = 0·148) (Table 1). In the multivariable Poisson model, compared with the NV, LOV and PV were 38 and 52 %, respectively, less likely to report DM, but these estimates were non-significant (Table 2). The zero diabetics among the VG made it impossible to calculate a PR. The self-reported prevalence of DM was supported by the mean fasting blood sugar levels among those not taking diabetic medication. Compared with a level of 5·24 mmol/l among the NV, the levels in the VG, LOV and PV were significantly lower at 4·77 mmol/l (*P* = 0·0007), 5·00 mmol/l (*P* = 0·004) and 4·91 mmol/l (*P* = 0·01), respectively, when not adjusting for BMI and this pattern remained when adjusting for BMI (Table 3).

### Serum lipids and dietary patterns

#### Serum total cholesterol

The proportion with high TC was lowest among the VG and LOV, with PR of 0·77 (95 % CI 0·55, 1·07) (*P* = 0·10) and 0·72 (95 % CI 0·59, 0·88) (*P* = 0·001), respectively, compared with the NV. These changed only modestly after also adjusting for BMI. The PV also tended to have lower PR at 0·87, but this did not reach statistical significance (Table 2). The lower prevalences of high TC were further supported by the mean TC levels among those not taking medication for hypercholesterolaemia where the VG and LOV had TC levels of 4·80 mmol/l (*P* = 0·05) and 4·75 mmol/l (*P* = 0·0002), respectively, compared with the 4·91 mmol/l among PV and 5·10 mmol/l among the NV (Table 3).

#### LDL-cholesterol and HDL-cholesterol

Similar to what was found for TC, both LDL-C and HDL-C also varied across the dietary patterns, with the PR of having high LDL-C ranging from 0·72 to 0·77 among all three vegetarian groups (Table 2) compared with the NV. When comparing the mean LDL-C levels, these were 3·0, 2·91 and 3·04 mmol/l, respectively, among the VG, LOV and PV, compared with 3·11 mmol/l among the NV, but only the findings for the LOV were statistically significant also when adjusting for BMI. Not unexpectedly, the prevalence of low HDL-C levels was similar across the dietary patterns (Table 2) as were the mean levels (Table 3) both with and without BMI in the model. Further sub-group analysis on adjusted means of HDL-C, however, showed that this difference was only present among female LOV compared with female NV after adjusting for BMI (1·32 *v.* 1·44 mmol/l, respectively; *P* = 0·01).

#### Non-HDL-cholesterol

Although the adjusted means of non-HDL-C were lower among all vegetarians than the NV irrespective of additional adjustment for BMI, only the levels among the LOV were significantly lower than the levels among the NV (3·53 *v.* 3·83 mmol/l; *P* = 0·002) and this only changed marginally when also adjusting for BMI (Table 3).

#### Serum TAG

The PR for high TAG were lower for all vegetarian groups compared with the NV (Table 2) and the corresponding mean TAG levels were also lower among the vegetarians (Table 3). However, only the mean among the LOV was statistically significantly lower (before adjusting for BMI) at 1·40 mmol/l (*P* = 0·03) (Table 3).

#### Total cholesterol:HDL-cholesterol, TAG:HDL-cholesterol and apoB:apoA-1

No significant association was observed between dietary patterns and mean TC:HDL-C or TAG:HDL-C ratio (Table 3). However, compared with the NV, the LOV had significantly lower apoB:apoA-1 ratio (0·71; *P* = 0·02) and this was very similar to levels among the VG and PV. These remained virtually unchanged when also adjusting for BMI.

### Obesity and abdominal adiposity and dietary patterns

Obesity and abdominal adiposity were strongly associated with dietary patterns by exhibiting lower PR of these risk factors among the vegetarian dietary patterns than among the NV (Table 2). The subgroup analysis showed that the PR among the females remained statistically significant for both of these among all vegetarian dietary patterns, whereas for males the PR reached statistical significance only among the LOV (Table 2). Compared with the NV where 32·8 % were obese, prevalence among the three vegetarian groups varied from 9·3 % among the VG, 11·1 % in the LOV and 10·5 % in the PV (*P* < 0·0001) (Table 1). Similarly, the adjusted mean WC values were 87·4, 90·5, 91·8, and 97·6 cm, respectively, for the VG, LOV, PV and the NV for both sexes combined (Table 3).

## Discussion

We found that vegetarians have lower CVD risk factor levels and less prevalent CVD than NV. VG tend to have lower risk factor levels than the other two types of vegetarians. Our findings were similar to those reported among the black Adventists, including lower OR of hypertension, dyslipidaemia, obesity and abdominal adiposity especially among the LOV/VG compared with the NV^(^^3^^)^. The only exception was a higher PR of low HDL-C among LOV than among NV among our white participants. The findings among the black AHS-2 participants were in general somewhat weaker than what we found among the white participants. However, among the blacks, there was no association between dietary patterns and BP, but a vegetarian diet was significantly associated with lower prevalence of DM. This could partly be due to the fact that the overall prevalence of DM was higher among the blacks than among the whites for all four dietary patterns (VG not reported *v.* 0·0 %, LOV/VG 8·9 *v.* 4·2 %, PV 18·8 *v.* 3·2 %, NV 15·6 *v.* 7·0 %, respectively). A long-term cohort study reporting consistently excessive energy intake in all diet groups among the blacks^(^^38^^)^ may partly explain the higher overall prevalence of DM (and as the National Vital Statistics Reports reported the higher ranking of DM as the leading cause of death among the blacks^(^^39^^)^) than among the whites which is also consistent with the finding of higher odds of class 3 obesity (BMI ≥ 40 kg/m^2^) both among black men and women than the non-Hispanic whites and other ethnic groups^(^^40^^)^. Among the whites, all three vegetarian groups had consistently lower FBG than the NV, whereas this was only the case for the LOV/VG and not the PV, among the blacks. The OR of prevalent DM among the PV was not lower than the NV in the blacks.

Our findings of lower FBG among vegetarians are also in line with what has previously been reported from another randomly sampled cross-sectional sub-study of AHS-2 where the LOV/VG had significantly lower FBG compared with the NV^(^^5^^)^. The adoption of various dietary patterns is a result of dietary preferences which may depend on both food availability and health consciousness. The adherence to a vegetarian diet seems to be moderately higher among the whites than among the blacks (62·9 *v.* 38·0 %^(^^3^^)^, respectively). This is in line with a recent retrospective study of the parent AHS-2 cohort where the great majority of life-time stable vegetarians >70 years of age were non-blacks^(^^41^^)^.

BMI as well as WC are each considered to be single independent risk factors for type 2 DM^(^^42^^–^^44^^)^. Significantly lower BMI and WC were seen among white and also among black vegetarians^(^^3^^)^ when compared with NV. In line with this finding, a reduced DM incidence was found among the black vegetarians (OR 0·30–0·47) compared with the NV 2 years after AHS-2 participants completed the baseline FFQ^(^^45^^)^. However, the blacks had higher odds of DM than the whites (OR 1·36, 95 % CI 1·09, 1·70)^(^^45^^)^. Another longer follow-up of the AHS-2 found a lower hazard ratio (HR) of fatal DM (HR = 0·53, 95 % CI 0·32, 0·89) among vegetarians compared with NV^(^^46^^)^. Two previous studies among AHS-2 participants have found that vegetarians have lower prevalence of both DM^(^^6^^)^ and the metabolic syndrome^(^^5^^)^ after adjusting for a number of co-variables including race/ethnicity. Thus there may be a potential protective association of vegetarian dietary patterns with DM independent of race^(^^5^^)^, in part, possibly mediated by obesity and fat distribution^(^^47^^)^.

The Mediterranean diet is in many ways similar to a vegetarian diet in its distinguishing characteristics such as being high in vegetables, fruits, legumes, nuts, grains, unsaturated fatty acid:SFA ratio, and low in meat and meat products^(^^48^^,^^49^^)^. The strength of association between such a diet and DM is similar to our findings. The potential mechanistic pathway leading to lower CVD risk among vegetarians compared with NV may, in part, be explained by cardioprotective dietary intake with a more favourable fat intake profile (low SFA and total fat, but high PUFA), lower total energy, lower intakes of protein and Na, but higher intakes of dietary fibre, vitamins C, E and B_1_, folate, Mg and Fe among VG and LOV compared with NV^(^^33^^,^^50^^)^. In fact, similar favourable nutrient contents among vegetarian dietary patterns were observed in a separate sub-study of AHS-2^(^^23^^)^. Another separate sub-study reported a higher consumption of nuts and seeds among the vegetarian dietary patterns compared with the NV in the AHS-2^(^^51^^)^ and the vegetarian dietary patterns were associated with reduced CVD risk^(^^52^^,^^53^^)^. The VG and LOV had higher healthy dietary indices (Healthy Eating Index (HEI2010) and Mediterranean Diet Score (MDS)) than NV in a cross-sectional online survey^(^^54^^)^. The HEI2010 has been shown to be inversely associated with CVD risk factors^(^^14^^)^ and CVD mortality^(^^15^^)^. When comparing adherence to the Mediterranean diet using the MDS^(^^48^^)^ as an indicator of adherence to this diet, a Danish cohort study found an inverse association between the score and CVD^(^^55^^)^. Those with a one-unit increase in the score had 6, 10, 11 and 20 % lower risk of incident CVD, fatal CVD, incident myocardial infarction (MI) and fatal MI, respectively. For stroke, there was a 4 % lower incidence, but a non-significant 3 % higher mortality with one-unit score increase^(^^55^^)^. The UK-based EPIC-Norfolk cohort study also found that the degree of adherence to the Mediterranean diet (each 1 sd increase in the score) was associated with 5 and 9 % lower incidence and mortality of CVD, respectively^(^^56^^)^. A similar 17 and 21 % protective effect in the highest quintile *v.* the lowest quintile of the MDS, and energy-adjusted MDS, respectively, was reported for CVD mortality in a large multi-ethnic cohort (*n* 193 527) in the USA^(^^57^^)^. In a 10-year follow-up of a Greek population, a significant inverse association was reported between the MDS and fatal or non-fatal CVD incidence as well as with five principal CVD risk factors including (1) anthropometric and (2) lipid profiles, (3) BP, (4) glucose profile and (5) inflammatory factors^(^^58^^)^. Another prospective cohort study consisting of Czech, Polish and Russian participants (*n*  19 263) reported that 2·2 points or 1 sd increase in the MDS was associated with a significant 10 % reduction in CVD mortality (438 deaths), a 10 % non-significant reduction in IHD, and a 13 % reduction in stroke mortality (226, and 109 deaths, respectively)^(^^59^^)^.

Similar findings were reported from a 24-year follow-up of middle-aged female nurses of the Nurses’ Health Study cohort on the Dietary Approaches to Stop Hypertension (DASH)-style diet, which encouraged the intake of fruits, vegetables, nuts and legumes, whole grains, and low-fat dairy products while discouraging Na, red and processed meats, and sweetened beverages^(^^60^^)^. Among women with a history of hypertension, physical activity less than the median, or smokers, the effect of the DASH diet was stronger than among those without these characteristics, with relative risks (RR) of (with/without) 0·68/0·76, 0·65/0·83 and 0·65/0·80, respectively, for CHD incidence associated with the highest *v.* the lowest quintile of the DASH score^(^^60^^)^. This was also the case for incident stroke among these subgroups, with RR of (with/without) 0·64/0·87, 0·88/0·70 and 0·67/0·90, respectively^(^^60^^)^.

The EPIC-Oxford cohort study, similar to findings from the AHS-2 study^(^^46^^)^, also reported on the benefits of a vegetarian diet, with HR of IHD death being 17 % lower among vegetarians compared with NV^(^^61^^)^.

Non-HDL-C is expressed as the difference between TC and HDL-C, and is considered to be an IHD risk indicator avoiding confounding from LDL and TAG^(^^62^^)^. We found a lower adjusted non-HDL-C (TC minus HDL-C) level ranging from 3·53 to 3·66 mmol/l in the three vegetarian dietary patterns compared with 3·84 mmol/l among the NV which remained approximately the same after also adjusting for BMI. These levels are similar to those reported by the newer EPIC-Oxford cohort study where adjusted means were 4·42 (95 % CI 4·36, 4·47) and 3·97 (95 % CI 3·84, 4·10) mmol/l for NV (*n* 1316) and vegetarians (*n* 230), respectively^(^^9^^)^. In line with this, their study reported 32 % lower HR of fatal or non-fatal IHD among vegetarians compared with the NV, very similar to our findings of 29 % lower hazard of fatal IHD among vegetarian men and 12 % lower among vegetarian women compared with the NV AHS-2 participants^(^^46^^)^.

Low HDL-C levels were previously (i.e. the Adult Treatment Panel (ATP) III guidelines) considered ‘an independent risk factor for CHD’^(^^63^^)^. Despite the fact that ATP IV guidelines no longer recommended specific levels of lipoproteins^(^^64^^)^, it maintained a tool to estimate 10-year risk of atherosclerotic CVD, referred to as the CV Risk Calculator, requiring inputs of TC and HDL-C levels as well as other information^(^^65^^)^. While a low HDL-C may still be a concern^(^^66^^)^ especially among vegetarians, it has also been known that a low-fat vegetarian diet reduces HDL-C^(^^67^^–^^69^^)^. A review suggested that the TC:HDL-C ratio has a greater predictive value as well as higher specificity of the risk of CVD than either one of these factors alone^(^^70^^)^. A Swedish cross-sectional study equated the ability of TC:HDL-C to indicate dyslipidaemia with that of apoB:apoA-I^(^^71^^)^. Although a prospective case–control study among apparently healthy participants nested within the EPIC-Norfolk study did not report directly the TC:HDL-C ratio, approximate values of it can be derived from their univariate analysis as 5·12 and 4·57 for IHD death cases and controls, respectively^(^^72^^)^. The values of the present study in all dietary patterns were lower than seen in the EPIC-Norfolk controls. On the other hand, the EPIC-Oxford cohort study reported the TC:HDL-C ratios for NV (4·58, 95 % CI 4·51, 4·66) and vegetarians (4·39, 95 % CI 4·21, 4·56)^(^^9^^)^. The values of the present study in all dietary patterns were lower than seen in the NV of the EPIC-Oxford cohort. However, our ratios oscillated around the value seen in the vegetarians of the said cohort^(^^9^^)^.

The TAG:HDL-C ratio has also been suggested as a strong predictor of atherogenesis and hence CVD^(^^73^^–^^76^^)^. However, we found no significant association of the ratios with the dietary patterns. The apoB:apoA-I ratio has been proposed to have a better association with IHD than other lipid ratios^(^^36^^,^^77^^)^ and better than LDL-C alone^(^^78^^)^ and has been used as a potential plasma atherogenic marker^(^^79^^)^. In the present study, the LOV had the lowest ratio, 0·71 among the vegetarians, 0·04 units lower than that among the NV. The apoB:apoA-I ratio has been reported to have the strongest predictability of coronary risk when LDL-C < 3·6 mmol/l^(^^80^^)^. Having lower LDL-C than this cut-off level among all vegetarian dietary patterns, and considering the fact that the HR of CVD death among the vegetarians in the parent cohort were lower than the NV^(^^46^^)^, a reduction as small as 0·04 in the apoB:apoA-I ratio may be of biological importance. Although the regression-generated ratios may not be directly applicable to the US population, the tendency of the lower ratios among the vegetarians compared with the NV along with the lower PR of CVD risk factors among the vegetarians support the possibility that the vegetarians with low HDL would potentially have lower risk when LDL was also low. However, it should also be cautiously interpreted due to confounding, and whether a small difference in such a ratio truly reflects an important clinical implication is not clear.

In the present study, the PV tended to be quite similar to the VG and the LOV with respect to prevalence of risk factors and their adjusted mean levels. This was also the case among the black AHS-2 participants^(^^3^^)^ and in line with the findings from a follow-up study of the AHS-2 parent cohort^(^^46^^)^.

The limitation of the present study includes the fact that the clinical data were obtained 1–3 years after the dietary pattern classification. Thus, there could be some misclassification and thus attenuation of associations. The lower, but non-significant, findings of PR for lipid profiles, obesity and abdominal adiposity among VG and PV are most probably due to relatively low numbers of participants in these groups resulting in low power. Another limitation is that any diagnosis of CVD before enrolment into the AHS-2 could have resulted in changes in dietary patterns and thus attenuation of the associations between dietary patterns and CVD risk factors (reverse causation). It is also known that AHS-2 participants tend to change their diet to a more plant-based one as they age^(^^41^^)^. This could also result in diluting the contrast between the vegetarians and the NV, with respect to PR and mean levels of CVD risk factors^(^^81^^)^. In spite of these possibilities, the present study did not exclude these prevalent cases because we wanted to be able to examine the association between dietary patterns and prevalent CVD risk factors as well as their mean levels. Our study could also suffer from selection bias by healthy participants and measurement error in dietary pattern determination at baseline^(^^45^^)^ and both of these would weaken the association between dietary patterns and CVD risk factors. Nevertheless, the observed association of the baseline dietary patterns with the CVD risk factor levels at the subsequent clinics suggests that, in spite of these possible factors, the association is robust and possible misclassification is relatively small^(^^41^^)^, as suggested by the fact that the difference in dietary pattern classification between the baseline AHS-2 and the calibration study was non-significant^(^^21^^)^.

The consistency of the present study finding with those of non-AHS-2 studies and other AHS-2 sub-studies strengthens the conclusions of our findings that there is a clear association between dietary patterns and CVD risk factors. The fact that we observed significant associations in spite of a potential conservative bias might imply that the true associations are even stronger.

### Conclusion

In summary, our study findings are in line with other studies reporting that cardiovascular risk factors are more favourable among participants following a vegetarian dietary pattern compared with those who are NV. The vegetarian dietary patterns were associated with lower prevalence of CVD risk factors compared with the NV. Although attenuated, this association remained after adjusting for BMI especially among the LOV and the VG. Even though CVD risk factor levels varied between the three different vegetarian dietary patterns, these differences were attenuated after adjusting for BMI, but still the estimates remained lower than those found among the NV. Our findings are compatible with recent findings of lower risk of fatal CVD among AHS-2 vegetarians compared with NV. The findings also show that the inverse association with fatal CVD is similar across the three types of vegetarian dietary patterns. Further research is needed on how to effectively reach the population at large to promote plant-based dietary patterns as protective in reducing CVD. Also, additional longitudinal studies with higher number of participants in the various vegetarian diet groups are needed to more clearly determine which types of vegetarian dietary patterns, considering also the food quality of each, are the most effective in reducing CVD.
